# Diagnostic Use of Post-therapy ^131^I-Meta-Iodobenzylguanidine Scintigraphy in Consolidation Therapy for Children with High-Risk Neuroblastoma

**DOI:** 10.3390/diagnostics10090663

**Published:** 2020-09-02

**Authors:** Hiroshi Wakabayashi, Daiki Kayano, Anri Inaki, Raita Araki, Rie Kuroda, Norihito Akatani, Takafumi Yamase, Satoru Watanabe, Tomo Hiromasa, Yuji Kunita, Hiroshi Mori, Shintaro Saito, Yasuhiro Ikawa, Toshihiro Fujiki, Seigo Kinuya

**Affiliations:** 1 Department of Nuclear Medicine, Kanazawa University Hospital, 13-1 Takara-machi, Kanazawa, Ishikawa 920-8641, Japan; kayano@staff.kanazawa-u.ac.jp (D.K.); henri@staff.kanazawa-u.ac.jp (A.I.); akatani@staff.kanazawa-u.ac.jp (N.A.); tyamase@med.kanazawa-u.ac.jp (T.Y.); watanabe@nmd.m.kanazawa-u.ac.jp (S.W.); hiromasat@staff.kanazawa-u.ac.jp (T.H.); kunita.nmd@gmail.com (Y.K.); hirmri@staff.kanazawa-u.ac.jp (H.M.); shintaro1515@stu.kanazawa-u.ac.jp (S.S.); kinuya@med.kanazawa-u.ac.jp (S.K.); 2 Department of Pediatrics, Kanazawa University Hospital, 13-1 Takara-machi, Kanazawa, Ishikawa 920-8641, Japan; raraki@staff.kanazawa-u.ac.jp (R.A.); pedkuro@staff.kanazawa-u.ac.jp (R.K.); yasuhiro.ikawa@staff.kanazawa-u.ac.jp (Y.I.); fujiki@staff.kanazawa-u.ac.jp (T.F.)

**Keywords:** diagnostic ^123^I-mIBG scintigraphy, post-therapeutic ^131^I-mIBG scintigraphy, high-risk neuroblastoma, consolidation therapy

## Abstract

^123^I-meta-iodobenzylguanidine (^123^I-mIBG) scintigraphy is used for evaluating disease extent in children with neuroblastoma. ^131^I-mIBG therapy has been used for evaluation in children with high-risk neuroblastoma, and post-therapy ^131^I-mIBG scintigraphy may detect more lesions compared with diagnostic ^123^I-mIBG scintigraphy. However, no studies have yet revealed the detection rate of hidden mIBG-avid lesions on post-therapy ^131^I-mIBG whole-body scan (WBS) and SPECT images in neuroblastoma children without mIBG-avid lesions as demonstrated by diagnostic ^123^I-mIBG scintigraphy. We retrospectively examined the diagnostic utility of post-therapy ^131^I-mIBG scintigraphy in children who received ^131^I-mIBG as consolidation therapy. Nineteen children with complete response to primary therapy were examined. Post-therapy ^131^I-mIBG scintigraphy was performed four days after injection. The post-therapy ^131^I-mIBG scintigraphy, 4 children exhibited abnormal uptake on the WBS. Post-therapy ^131^I-mIBG SPECT/CT provided additional information in 2 cases. In total, 6 children exhibited abnormal uptake. The site of abnormal accumulation was on the recurrence site in one case, operation sites in five cases, and bone metastasis in one case. Post-therapy ^131^I-mIBG scintigraphy could detect residual disease that was not recognized using diagnostic ^123^I-mIBG scintigraphy in 32% of children with high-risk neuroblastoma and ganglioneuroblastoma. The diagnostic use of post-therapy ^131^I-mIBG scintigraphy can provide valuable information for detecting residual disease.

## 1. Introduction

^123^I-meta-iodobenzylguanidine (^123^I-mIBG) scintigraphy has been used for evaluating disease extent in children with neuroblastoma, and ^131^I-mIBG therapy has been used for children with high-risk neuroblastoma and mIBG-avid lesions [1,2,3]. Consolidation therapy incorporating high-dose ^131^I-mIBG has yielded good overall and event-free survival in children with high-risk neuroblastoma with complete response (CR) to primary therapy as demonstrated by diagnostic ^123^I-mIBG scintigraphy [4,5]. 

The accurate evaluation of the remaining mIBG-avid lesions on diagnostic ^123^I-mIBG scintigraphy is important for deciding sites of the tumor bed, gross residual disease, and mIBG-avid bone metastases, which are to receive external radiation therapy in consolidation therapy [6,7,8]. Post-therapy ^131^I-mIBG scintigraphy may detect more lesions compared with diagnostic ^123^I-mIBG scintigraphy in children with neuroblastoma [9]. This may be due to different kinetics, the presence of a higher amount of carriers, and delayed scanning time [10,11,12]. Patients with sustained CR had residual avid lesions in post-therapy ^131^I-mIBG scan as demonstrated in the ^123^I-mIBG scintigraphy. Johnson et al. demonstrated that five of five relapsed/refractory neuroblastoma patients with CR by diagnostic ^123^I-mIBG scintigraphy showed abnormal uptake in post-therapy ^131^I-mIBG whole-body scan (WBS) images [13]. Suh et al. demonstrated that three of fifteen newly diagnosed neuroblastoma patients with CR by diagnostic ^123^I-mIBG scintigraphy showed abnormal uptake in post-therapy ^131^I-mIBG images [5]. The percentage varies depending on the study; high-risk neuroblastoma patients have hidden mIBG-avid lesions, which is under the level of detection by diagnostic ^123^I-mIBG images.

Post-therapy ^131^I-mIBG single-photon emission computed tomography (SPECT)/ computed tomography (CT) images can provide additional diagnostic information over planar images [14]. However, no studies have yet revealed the detection rate of hidden mIBG-avid lesions in post-therapy ^131^I-mIBG SPECT/CT in children with high-risk neuroblastoma with CR to primary therapy as demonstrated by diagnostic ^123^I-mIBG scintigraphy. Herein, we retrospectively examined the diagnostic utility of post-therapy ^131^I-mIBG scintigraphy using WBS and ^131^I-mIBG SPECT/CT images in children who received ^131^I-mIBG as consolidation therapy.

## 2. Materials and Methods

### 2.1. Ethical Considerations and Registration

This study was conducted in accordance with the Declaration of Helsinki and the International Committee for Harmonization of Good Clinical Practice guidelines. The Institutional Review Board of Kanazawa University Hospital approved the study (UMIN000002530, the approval date was September 11 in 2009 and UMIN000025045, the approval date was December 1 in 2016). Written informed consent was obtained in writing from participants and/or their guardians prior to registration.

### 2.2. Inclusion Criteria

The study’s inclusion criteria were as follows: (1) patients who were either newly diagnosed or had relapsed to high-risk neuroblastoma in accordance with Children’s Oncology Group (COG) or International Neuroblastoma Risk Group classification; (2) patients who had one or more lesions at initial presentation or relapse that accumulated ^123^I-mIBG; (3) patients who had no ^123^I-mIBG avid lesion at ^131^I-mIBG therapy; (4) an availability of cryopreserved autologous peripheral blood stem cells, cord blood, or other stem cell sources; (5) patients who had adequate organ function and a life expectancy of at least 3 months; and (6) patients with estimated glomerular filtration rate more than 30 mL/min/1.73m^2^.

From December 2009 to March 2019, 19 children had CR to primary therapy as determined by diagnostic ^123^I-mIBG scintigraphy. The disease stage was determined according to the International Neuroblastoma Staging System (INSS). The Children’s Oncology Group (COG) presently defines high-risk neuroblastoma as (1) Stage 2A or 2B disease with *MYCN* amplification, (2) Stage 3 disease with *MYCN* amplification, (3) Stage 3 disease in children aged 18 months or older with no *MYCN* amplification but unfavorable histopathology, (4) Stage 4 disease in children aged younger than 12 months with *MYCN* amplification, (5) Stage 4 disease in children aged 12–18 months with *MYCN* amplification and/or diploidy and/or unfavorable histology, (6) Stage 4 disease in children aged 18 months or older, or (7) Stage 4S disease with *MYCN* amplification.

### 2.3. Treatment

^131^I-mIBG therapy was planned on the basis of draft guidelines on the appropriate use of ^131^I-mIBG radiotherapy for neuroendocrine tumors by the Guideline Drafting Committee for Radiotherapy with ^131^I-mIBG, the Committee for Nuclear Oncology and Immunology, the Japanese Society of Nuclear Medicine, and the procedure guidelines for ^131^I-mIBG therapy from the European Association of Nuclear Medicine (EANM) [2,15]. In a radiation isolation room of Kanazawa University Hospital, all children received ^131^I-mIBG (Izotop, Budapest, Hungary, from December 2009 to January 2012, and POLATOM, Otwock, Poland, from November 2012 to March 2019) therapy without acute severe adverse effects.

### 2.4. Imaging

Before ^131^I-mIBG treatment, ^123^I-mIBG WBSs and combined SPECT/CT (SymbiaT6, Siemens Medical Solutions, Tokyo, Japan, from December 2009 to March 2016, and Discovery NM/CT 670 Q. Suite Pro, GE Healthcare, Tokyo, Japan, from April 2016 to March 2019) were performed 24 h after intravenous injection of ^123^I-mIBG (FUJIFILM RI Pharma Co., Ltd., Tokyo, Japan). Anterior and posterior WBSs were performed at a speed of 12 cm/min using a 256 × 1024 matrix and a 159-keV photopeak with a 15% window. SPECT data were acquired with a 128 × 128 matrix and 60 projections (20 s per view). Following SPECT data acquisition, CT transmission scanning for topography was performed. SPECT and CT were acquired during shallow breathing with the patient lying quietly in the supine position. The interval between SPECT and CT was of a few minutes. SPECT was reconstructed using a three-dimensional ordered-subset expectation maximization algorithm with resolution recovery, scatter correction, and CT-based attenuation correction. SPECT and CT data were analyzed and co-registered using a workstation (Syngo Acquisition Workplace for SymbiaT6 and Xeleris 3.1 for Discovery NM/CT 670 Q. Suite Pro).

Post-therapy ^131^I-mIBG WBSs and SPECT/CT were performed four days after injection with ^131^I-mIBG. Anterior and posterior WBSs were performed at a speed of 15 cm/min using a 256 × 1024 matrix and a 364-keV photopeak with a 20% window. SPECT data were acquired with a 128 × 128 matrix and 60 projections (20 s per view). SPECT and CT were reconstructed in the same way as ^123^I-mIBG scintigraphy. Board-certified physicians of nuclear medicine with 9, 13, and 16 years of experience reviewed WBSs and SPECT/CT images on both of ^123^I-mIBG and ^131^I-mIBG scintigraphy for detecting residual disease with clinical information.

### 2.5. Statistical Analysis

Results are expressed as the mean ± standard deviation. Statistical analyses were performed using JMP software (Version 14.0.0, SAS Institute Inc., Cary, NC, USA). Differences in abnormal uptake between ^123^I-mIBG and ^131^I-mIBG scintigraphy were calculated using student’s *t*-testing. A *p*-value of <0.05 was considered statistically significant.

## 3. Results

There were 18 children with high-risk neuroblastoma and one child with high-risk ganglioneuroblastoma with CR to primary therapy in diagnostic ^123^I-mIBG scintigraphy (sex: male = 7 and female = 12; age: 7.4 ± 3.8 years (range: 1–16)). Nine children were initially diagnosed (*n* = 9; age, 5.9 ± 4.7 years), and 10 children had recurrent neuroblastoma (*n* = 10; age: 8.8 ± 1.9 years). Two children had stage III disease and 17 had stage IV disease according to the INSS. *MYCN* amplification was positive in 11 children and negative in 8 children.

The administration dose of ^131^I-mIBG was as follows: high-dose (666 MBq/kg, *n* = 16), and intermediate-dose (444–555 MBq/kg, *n* = 3). The dose of ^131^I-mIBG was reduced to intermediate in two children due to the upper limit of radioisotope use in our university hospital. One child received a reduced ^131^I-mIBG dose due to decreased renal function and was categorized as an intermediate dose of ^131^I-mIBG.

**Figure 1:**
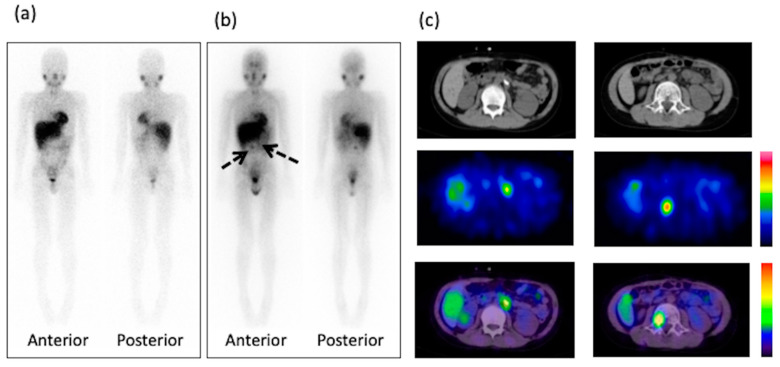
Eleven-year-old male with high-risk neuroblastoma. (**a**) Whole-body ^123^I-mIBG scintigraphy one day after injection shows no abnormal uptake. (**b**) Whole-body ^131^I-mIBG scintigraphy four days after injection shows definite abnormal uptake (arrows). (**c**) CT, SPECT, and fused SPECT/CT images demonstrate the uptake at the operation site and at L3. mIBG, meta-iodobenzylguanidine; SPECT, single-photon emission computed tomography; CT, computed tomography; L3, third lumber vertebra.

**Figure 2:**
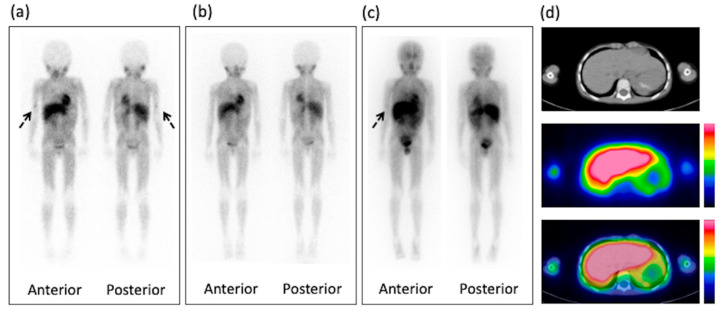
Six-year-old male with high-risk neuroblastoma. (**a**) Whole-body ^123^I-mIBG scintigraphy one day after injection shows abnormal uptake on the right humerus at relapse (arrows). (**b**) The uptake disappears before ^131^I-mIBG therapy on the whole-body ^123^I-mIBG scintigraphy. (**c**) Whole-body ^131^I-mIBG scintigraphy four days after injection shows faint uptake at the relapse site on the right humerus (arrow). (**d**) CT, SPECT, and fused SPECT/CT images demonstrate the uptake at the relapse site on the right humerus. mIBG, meta-iodobenzylguanidine; SPECT, single-photon emission computed tomography; CT, computed tomography.

On post-therapy ^131^I-mIBG scintigraphy, 4 of 19 children (21%) exhibited definite abnormal uptake on the WBS. Post-therapy ^131^I-mIBG SPECT/CT provided additional information in 2 of the 19 children (11%), which was the presence of suspicious findings on WBS images. A significant difference was noted in the number of abnormal uptakes between ^123^I-mIBG (0/19, 0%) and post-therapy ^131^I-mIBG (6/19, 32%) scintigraphy (*p* = 0.0075). The representation of this abnormal uptake is shown in Figure 1; Figure 2. The site of abnormal accumulation was at the recurrence site in one case, at operation sites in five cases, and at the bone metastasis site in one case. The diagnosis differed between readers in 1 case (5%), and they discussed and decided the score in mutual agreement.

## 4. Discussion

Post-therapy ^131^I-mIBG scintigraphy could detect residual mIBG-avid lesions that were not detected using diagnostic ^123^I-mIBG scintigraphy in children with high-risk neuroblastoma and ganglioneuroblastoma.

Post-therapy ^131^I-mIBG scintigraphy detected one or two occulted mIBG-avid lesions in 32% of patients with neuroblastoma in the present study. Post-therapy ^131^I-mIBG SPECT/CT was useful to detect two mIBG-avid lesions, which correlated with the presence of suspicious findings on WBS images. Our data was compatible with data from previous studies demonstrating improved lesion detectability with post-therapy ^131^I-mIBG scintigraphy compared with that of diagnostic ^123^I-mIBG scintigraphy in patients with neuroblastoma with mIBG-avid lesions. For patients with neuroblastoma with mIBG-avid lesions in the diagnostic ^123^I-mIBG scintigraphy, Yang et al. demonstrated that post-therapy ^131^I-mIBG scintigraphy revealed additional lesions compared with ^123^I-mIBG scintigraphy (total mIBG-avid lesions: 716 vs. 532 lesions) in 56 patients (44%) [16]. Hickeson et al. showed that 210 lesions were detected on the post-therapy ^131^I-mIBG scintigraphy compared with 151 on the ^123^I-mIBG scintigraphy in 16 patients (89%) [17]. For the patients with neuroblastoma without mIBG-avid lesions on diagnostic ^123^I-mIBG scintigraphy, post-therapy ^131^I-mIBG WBS images detected occult lesions in five of five (100%) [13] and post-therapy ^131^I-mIBG scan with no information about SPECT in three of fifteen (20%) patients [5]. This study found that the detection rate of 32% in patients without mIBG-avid lesions was less compared to that in most reports on patients with mIBG-avid lesions using ^123^I-mIBG scintigraphy.

The accurate evaluation of remaining mIBG-avid sites is critical in deciding the metastatic site. External irradiation is superior to local control of the primary site and mIBG-avid sites present after induction chemotherapy and resection [18,19]. Patients with few mIBG-avid lesions have a higher chance of being treated using external radiotherapy. In our study, one patient had occulted mIBG-avid bone metastasis, and five patients had occulted mIBG uptake in the primary site. This occulted uptake reflecting tumor activity might be important in planning total dose administered, fractionation, and field arrangement of external radiation. In the current study, following ^131^I-mIBG therapy, most patients returned to their referring hospitals to receive different additional following treatment, including high-dose chemotherapy, hematopoietic stem-cell transplantation, maintenance therapy, surgery, and external radiation therapy, according to the judgment of the attending physician. We could not determine which additional treatment was required for the remaining mIBG-avid sites in the current study. Further studies are needed for investigating how the additional information yielded by post-therapy ^131^I-mIBG scintigraphy in patients without ^123^I-mIBG-avid lesions affects relapse-free survival.

^131^I-mIBG as consolidation therapy can be used for diagnostic and therapeutic purposes. As a therapeutic option, we propose high-dose ^131^I-mIBG therapy as consolidation therapy with present multimodality treatments. However, our study was retrospective in design, and the number of patients was small; thus, a multicenter prospective study is warranted for confirming the utility. Diagnostically, ^131^I-mIBG scintigraphy could confirm the activity of lesions that were not detected on diagnostic ^123^I-mIBG scintigraphy but were suspected with other imaging modalities, such as CT and magnetic resonance imaging. Accurate detection of remaining mIBG-avid lesions is clinically important to suggest which sites should be treated using external radiation therapy.

## 5. Conclusions

Post-therapy ^131^I-mIBG scintigraphy could detect residual disease that was not recognized by diagnostic ^123^I-mIBG scintigraphy in 32% of children with high-risk neuroblastoma and ganglioneuroblastoma. Post-therapy SPECT/CT was useful to detect mIBG-avid lesions, which correlated with presence of suspicious findings on WBS images. The diagnostic use of post-therapy ^131^I-mIBG scintigraphy can provide valuable information for detecting residual disease.
